# Improving the Pedestrian’s Perceptions of Safety on Street Crossings. Psychological and Neurophysiological Effects of Traffic Lanes, Artificial Lighting, and Vegetation

**DOI:** 10.3390/ijerph17228576

**Published:** 2020-11-19

**Authors:** Carmen Llinares, Juan Luis Higuera-Trujillo, Antoni Montañana, Nuria Castilla

**Affiliations:** 1 Institute for Research and Innovation in Bioengineering (i3B), Universitat Politècnica de València, 46022 Valencia, Spain; cllinare@omp.upv.es (C.L.); amonav@doe.upv.es (A.M.); 2 Escuela de Arquitectura, Arte y Diseño (EAAD), Tecnologico de Monterrey, Monterrey 64849, Mexico; 3 Department of Architectural Constructions, Universitat Politècnica de València, 46022 Valencia, Spain; ncastilla@csa.upv.es

**Keywords:** pedestrian evaluation, urban design, neuro-architecture, virtual reality

## Abstract

The effect that the physical characteristics of urban design have on the pedestrian’s perceptions of safety is a fundamental aspect of city planning. This is particularly so with street crossings, where the pedestrian has to make a decision. This paper analyses how pedestrians are affected by number of traffic lanes, lighting colour temperature, and nearby vegetation as they cross roads. Perceptions of safety were quantified by means of the psychological and neurophysiological responses of 60 participants to 16 virtual reality scenarios (4 day and 12 night), based on existing urban design variables. The results showed differences between night-time and daytime scenarios, which suggests that there is a need to analyse both situations. As to the design guidelines, it was observed that safety is improved by reducing the number of traffic lanes and nearby vegetation, and by using a lighting colour temperature of 4500 K. However, the analysis of the variables showed that combined effects produce different results to those obtained from the analysis of individual elements. This result is essential information for urban managers in their assessments of whether particular interventions will improve crossing points.

## 1. Introduction

Perceptions of safety in urban spaces are fundamental for users of public roads. Perceptions of risk can influence decisions, such as the choice of pedestrian routes. Thus, how pedestrians perceive the risk associated with space is an important topic in the study of behaviour. To improve perceptions of safety, some studies have analysed the impact of urban space design variables and pedestrians’ responses to them [1].

One of the design variables traditionally analysed for this purpose is pedestrian infrastructure. Pedestrian infrastructure parameters, such as gradient, width and materials, have been studied [2]. For example, [3,4] found that wide, flat, non-slip and unobstructed pavements improve safety. It has also been shown that the more traffic lanes a road has, the lower the willingness of drivers to give way to pedestrians and the lower are perceptions of safety [5]. Other studies, such as those by [6,7], focused on traffic speed as a determinant variable of perceived safety. In this sense, [8] point out the need for traffic calming to be considered in the design of urban space to restrict the speed of vehicles. Thus, [9] found that a shared-space between pedestrians and drivers, eliminating the division of areas for each of them, caused a dense and slow traffic with consequent increase in safety, especially for vulnerable users.

Another design variable that influences the pedestrian’s perceptions of safety is lighting. The visibility of urban spaces varies between day and night. At night-time, street lighting improves visibility and provides orientation. It also contributes to the perceptions of comfort and safety of people outside after dark [10]. This is because people feel safest when they have a good overview of the space in which they are moving and if they feel they are supported by other users [11]. It has been found in the previous literature that outdoor lighting has a significant effect on how a space is perceived and on perceived security [12,13,14,15,16]. Therefore, lighting should be considered an essential design variable in urban design.

Nature is another design variable that, while it has not been analysed from a safety perspective, can have positive effects on the pedestrian. Humans have a predisposition to respond positively to natural environments as they consider them important for their survival [17]. A variety of studies have shown that, compared to environments with little or no natural features, exposure to natural environments reduces stress, anxiety, and positively influences well-being [18,19]. For example, [20] concluded that views of trees reduced stress and improved mood; and [20] found that when subjects viewed green areas they were less stressful, angry, depressed, and had improved mood and concentration. Specifically, in terms of design variables, [21] observed that rounded and conical trees generated more positive emotional responses.

The work done on the safety perceptions, comfort and protection of pedestrians has focused on walkability. These have provided a good understanding of pedestrian mobility [22,23]. However, the way in which the built environment influences pedestrians’ behaviour at particularly important times, such as when crossing a road, has not been widely studied [24]. However, it has been found that pedestrians assess different design variables to calculate crossing risk; parked cars [25]; topographical aspects, such as the number of traffic lanes, speed ramps, street width [26,27,28]; whether there are traffic islands in the middle of the road [29]; traffic signal countdown timers at street crossings [30]; nature; the spatial distribution of buildings; and pedestrian and traffic density [31]. It is necessary to examine all these aspects, in which pedestrians interact with their surroundings, to understand this complex decision-making process [32].

However, a general limitation of these works is that the analyses of the design variables were carried out in isolation, so that the urban space, as a whole, was not taken into account. Since real spaces combine numerous design variables, this can generate a problem for design guidelines; it is possible that the results of analyses of individual elements will not be reflected in analyses of a combination of elements. In addition, the results of analyses where design variables are combined are of greater interest in instances where existing urban spaces need to be improved. Thus, for example, vegetation can create a certain perception during the day, but a totally different one at night; its influence might also be affected by road infrastructure, such as the number of traffic lanes. This may mean that design guidelines are not universal but depend on a combination of variables that have influence at a given time.

This issue creates a need to simulate scenarios; when carrying out experiments in real physical spaces it difficult to modify some design variables while keeping others unchanged. Hitherto, studies have been carried out using photographs and videos [17,25,33,34]. These formats have limitations, such as the requirement to use a predefined viewpoint and lack of interactivity. To the extent that a stimulus is detached from reality, the results obtained are distorted [35]. In this respect, virtual reality is proposed as an effective alternative that can simulate scenarios; it offers the potential to conduct experiments which generate a sense of presence, or of “being there” [36], in an immersive, interactive simulation.

On the other hand, it is important to emphasize that the usual way of gathering pedestrians’ perceptions of safety is through self-report. A “dimensional” approach has often been used, that is, in the portrayal of the full spectrum of human emotions through independent dimensions. Among these approaches, the PAD (pleasure, arousal, dominance) emotional state model [37] should be highlighted. Perceptions of safety are integrated into dominance, understood as the feeling of control of space and the non-restriction of behaviours [38]. This model, in general, and dominance, in particular, has been applied in studies of environmental psychology, architecture and/or urban planning in the evaluation of the design variables in public spaces [39]. However, this way of quantifying perceptions of safety has limitations in that it may be biased by the respondent’s interactions.

This creates the need for a more complete quantification that includes unconscious processes related to the experience of urban space. As there are currently no neurophysiological metrics capable of quantifying pedestrians’ perceptions of safety in urban spaces, a more complete analysis of subjects’ responses requires the use of both psychological and neurophysiological variables. This hybrid approach allows conscious and unconscious components to be assessed, addressing the issue quickly and efficiently [40,41], and is compatible with the use of virtual reality technologies [42].

With this in mind, the purpose of this paper is to analyse the impact that design variables have on the pedestrian’s perceptions of safety, identifying the individual and combined effect of these variables. We selected three characteristic aspects of urban spaces previously discussed, one of them related to nature, another related to infrastructures, and finally, two variables related to lighting, natural and artificial, considering in the latter case the colour temperature. To simulate the isolated effect of these variables, virtual reality has been used. To obtain a more complete quantification, the issue was addressed by collecting pedestrians’ responses at both psychological and neurophysiological levels. The study focuses on street crossings in urban settings because they are fundamental aspects of the pedestrian’s perceptions of safety that, to date, have not been studied in depth.

## 2. Materials and Methods

**Figure 1:**
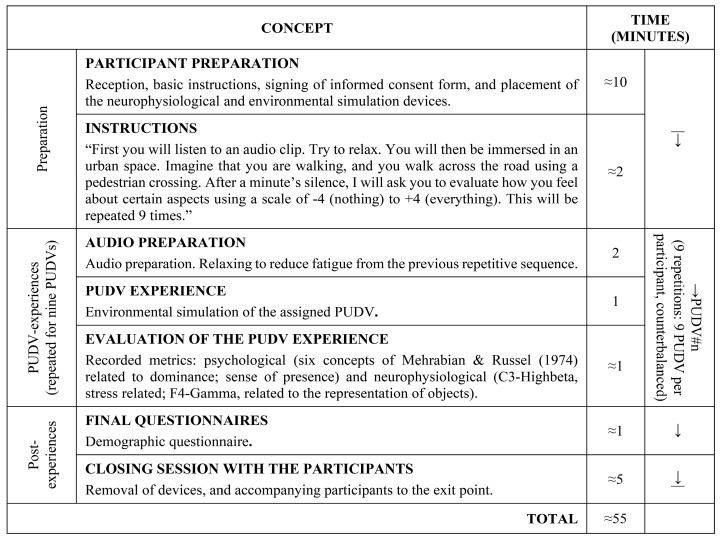
General testing sequence.

A field study was undertaken to examine participants’ perceptions of safety; different parameterizations of urban design variables (PUDV) were used. Figure 1 shows the general sequence.

Various analyses were performed on the collected data. First, the virtual simulations of the PUDVs were validated through participants’ level of presence (Analysis A). Throughout the participants’ PUDV experiences, psychological and neurophysiological metrics recorded the impact of the PUDVs on the participants’ general perceptions of safety (Analysis B), based on their profile (Analysis C). Subsequently, an examination was made of the differences between daytime and night-time simulations (Analysis D), and possible design guidelines for urban interventions (Analysis E).

### 2.1. Participants

There was a total of 60 participants. The sample size was determined following the calculations of [43] to achieve the desired alpha and beta error levels, and in line with similar protocols [41,44]. The sample was also controlled by gender and age. By gender, it was evenly balanced (50% men and 50% women). The age range (56% < 25 years; 20% 26–45 years; 15% 46–65 years; and 9% > 65 years) featured people over 65 because they need special attention [3]. All participants gave their informed consent for inclusion before they participated in the study. The study was conducted in accordance with the Declaration of Helsinki, and the protocol was approved by the Review Board of the Institute for Research and Innovation in Bioengineering (Project SPIP2017-02220) before the start of the experimental phase of the work (December 4 2017).

### 2.2. Stimuli

**Figure 2:**
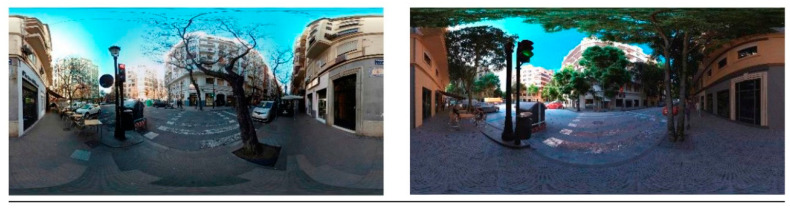
Comparison of the physical scenario (left) and its virtual replica (right; parameterization of urban design variable (PUDV) # B).

The base stimulus was a street crossing (immersive virtual replica, visual and auditory, of a real road intersection in the city of Valencia, Spain). This type of urban development is typical of the environmental expansion in the 19th century in this part of Europe. Figure 2 shows the comparison between the physical and virtual scenarios.

**Figure 3:**
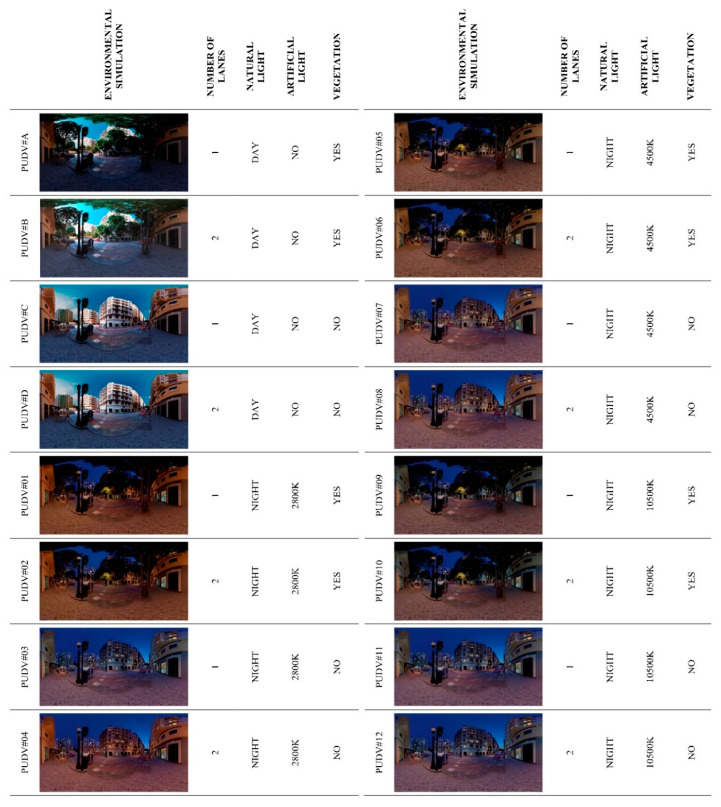
Configurations of the environmental simulations of the PUDVs.

The PUDVs were configured on the following basis. To guarantee the exhaustiveness of the study and that it is a work that can be addressed, four variables were studied: (1) “number of lanes”, (2) “natural lighting”, (3) “artificial lighting”, and (4) “vegetation”. The parameters for each were: number of lanes (1a) “1 lane”, and (1b) “2 lanes”; for natural lighting (2a) “day”, and (2b) “night”; for artificial lighting, colour temperatures of (3a) “2800 K”, (3b) “4500 K”, and (3c) “10,500 K”; and for vegetation (4a) “trees”, and (4b) “no-trees”. The combination of these resulted in 16 PUDVs (Figure 3).

All participants experienced nine different PUDVs. Of these, 3 were of natural light variable “day”, and 6 of natural light variable “night” (thus maintaining a consistent proportion of daytime and night-time PUDVs for all participants). The daytime experience PUDV # B (the base stimulus) was taken as a reference to normalize the psychological and neurophysiological metrics used in each case. Following this, all the experiences were randomized.

### 2.3. Set-Up of the Environmental Simulation

The participants’ PUDV experiences were evoked through visual and auditory environmental simulations. At a technical level, some aspects related to the generation of, and participants’ experience of, the PUDVs should be highlighted.

Regarding generation:

- At the visual level, the scenarios were modelled using the Rhinoceros system (v.5.0; www.rhino3d.com). They were rendered photo-realistically using Corona (v.2.0; https://corona-renderer.com), running on Autodesk 3ds Max (v.2014; www.autodesk.es). The resulting files were saved in jpg format, with a resolution of 8000 × 4000 pixels.
- At the auditory level, a binaural audio clip was generated. The recorder used was the digital ZOOM H4n Pro recorder (www.zoom-na.com), working with the Free Space XLR binaural microphone (www.3diosound.com). The corrections of the clips were made by Audacity (v.2.2.2; www.audacityteam.org). The resulting files were saved in 24-bit wav format, at 48,000 Hz.

The content for both was implemented in Unity3D (v5.6; www.unity3d.com).

Regarding the experience:

- At the visual level, HTC Vive glasses were used. This is a head-mounted display, from HTC and Valve (www.vive.com). It has a resolution of 1080 × 1200 pixels per eye (2160 × 1200 in total), with a field of view of 110°, and a refresh rate of 90 Hz.
- At the auditory level, HD 558 were used. These are headband earphones (headband type), by Sennheiser (www.en-us.sennheiser.com). They have a frequency response of 15 to 28,000 Hz.

### 2.4. Data Analysis

iMotions software was used to manage the protocol and compile the records. (v.6.1; www.imotions.com). For each participant, in addition to a basic demographic questionnaire (gender and age), psychological and neurophysiological data related to their perceptions of safety were recorded.

Psychological data. These were quantified through self-evaluation, on the one hand measuring dominance and, on the other, sense of presence during the PUDV environmental simulations. For dominance, several related concepts were used; and for presence, the SUS questionnaire was used.

- Dominance. To quantify dominance, the participants assessed the six descriptive [37] concepts (“controlling”, “influential”, “in control”, “important”, “dominant”, and “autonomous”) for each PUDV. A Likert-type scale of −4 to +4 was used.
- Presence. Sense of presence is the illusion of “being there” [36] in an environmental simulation. To quantify presence, the participants completed the SUS (after Slater, Usoh, and Steed) questionnaire [45]. This questionnaire consists of six items, assessed on a Likert scale, from 1 to 7. The objective was to verify that the simulations could be considered satisfactory.

Neurophysiological data. These were focused on quantifying complementary aspects related to the experience of urban space. Electroencephalogram metrics were used.

- The electroencephalogram (EEG) measures variations in the electrical activity of the surface of the scalp [46]. Two metrics were measured: the relative power (which reduces data variability; [47]) of the Highbeta band (21–30 Hz) of the C3 electrode, related to stress [48]; and the Gamma band (30–40 Hz) of the F4 electrode, related to the representation of objects [49].
  The b-Alert × 10 device (www.advancedbrainmonitoring.com) was used to record the electroencephalogram signals. The raw signal, sampled at 256 Hz, was pre-processed and analysed using the EEGLAB toolbox (v.14; https://sccn.ucsd.edu/eeglab) [50] through Matlab (v. 2016a; www.mathworks.com).
  The pre-processing consisted of two stages: (1) signal conditioning, and (2) artefact identification. The signal conditioning involved: (1) elimination of the baseline by subtraction of an average reference value; (2) filtration between 0.5 and 40 Hz [51]; (3) location of corrupted electrodes, considering them as such if the signal was flat for more than 10% of the duration of the recording, or if the kurtosis of the electrode reached a threshold of 5 standard deviations of the kurtosis of all electrodes [52]. Next, the signal was divided into one second epochs. The identification of artefacts involved: (1) location of corrupt epochs, considering them as such if their kurtosis reached the same threshold as the electrode scale; (2) automatic location, eliminating epochs that reached a threshold of 100 µV, or a gradient of 70 µV between epochs; and (3) application of independent component analysis (ICA) [53], rejecting those related to an artefact. A spectral classification analysis was performed on the pre-processed signal, using the Welch method, to calculate the selected metrics.

All metrics, with the exception of presence, were normalized in reference to the values obtained for PUDV#B (M_PUDV#x_ = (M_PUDV#x_ − |M_PUDV#B_|)/SD_PUDV#B_), where M is metric and SD refers to the standard deviation. In this way, the values of the metrics were displayed in relation to the parameterizations of the urban design variables.

### 2.5. Statistical Analysis

Following the collection and anonymization of the psychological and neurophysiological database, statistical analyses were carried out (Table 1). IBM SPSS software was used (v.17.0; www.ibm.com/products/spss-statistics). Statistical comparison techniques were mainly used (Analysis B, C, D, and E). To choose these techniques, the criterion of normality of the distribution of the data of the variable had to be verified using the Kolmogorov-Smirnov test (K-S test). This test determined that the variables did not meet the normality criterion (K-S Test, *p* < 0.05), so non-parametric statistical comparison techniques were used, such as Mann-Whitney, when the variable has two categories, and Kruskal -Wallis when it presents more than two categories.

## 3. Results

The statistical analysis of the data produced the following results.

### 3.1. Presence Level Analysis

**Figure 4:**
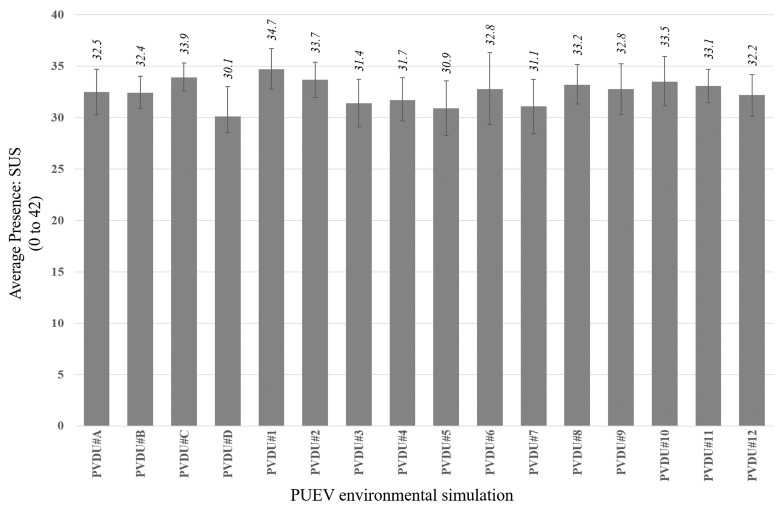
Average level at 95% confidence interval for presence mean in each PUDV simulation.

The average levels of sensation of presence per participant (according to the SUS questionnaire) for each environmental simulation were obtained (Figure 4). As the SUS questionnaire is made up of 6 items to be assessed on a 7-point Likert-type scale, the maximum presence level would be 42 points. They were considered to be sufficient, taking into account the results obtained by studies using similar technologies [54]. As such, the simulations can be considered satisfactory.

### 3.2. Analysis of the Impact of the PUDVs

Next, the differences in the results were examined based on number of traffic lanes, lighting colour temperature, and vegetation.

#### 3.2.1. Number of Traffic Lanes

To compare the metrics related to the perception of safety between the different PUDVs, taking into account that the variables do not meet the normality criterion (K-S test, *p* < 0.05), the Mann-Whitney test is applied due, which has two categories. The paired Mann–Whitney U tests detected significant differences in the dominance metric (*p* = 0.028) in the set of scenarios related to the number of traffic lanes variable. The highest level of dominance was shown in the case of fewer traffic lanes. At the neurophysiological level, the Mann–Whitney U tests found statistically significant differences for the stress-related metric C3-Highbeta (*p* = 0.010), with an increase in the mean rank attitude score with more traffic lanes. Greater effects were observed in the daytime scenarios (PUDV # AD) than in the night-time (PUDV # 1–12): in the daytime scenarios more traffic lanes led to a significant reduction in the dominance metric (*p* = 0.002), and a significant increase in the stress-related metric C3-Highbeta (*p* = 0.006) and the representation of objects in metric F4-Gamma (*p* = 0.000). In the night-time scenarios, more traffic lanes also caused a significant increase in the C3-Highbeta (*p* = 0.001) metric. Table 2 shows these results.

#### 3.2.2. Lighting Colour Temperature

To compare the metrics related to the perception of safety among the different PUDVs, taking into account that the variables do not follow a normal distribution (K-S Test, *p* < 0.05), the Kruskal-Wallis test is applied because variable lighting colour temperature has three categories. The Kruskal-Wallis test detected significant differences for the dominance metric (*p* = 0.000). The lowest level of dominance corresponded to a temperature of 2800 K, with a mean rank attitude score of 99.03. No significant differences were detected in the neurophysiological variables (*p* > 0.05). Table 3 shows these results.

#### 3.2.3. Vegetation

For the comparison of the metrics related to the perception of safety between the different PUDVs, the Mann-Whitney test is applied because it has two categories and the variables do not meet the normality criterion (K-S Test, *p* < 0.05). The paired Mann–Whitney U tests did not detect significant differences (*p* > 0.05) in the set of scenarios related to the vegetation variable, neither at the psychological level nor at the neurophysiological level. The only differences observed in this variable were between the daytime (PUDV # A–D) and night-time (PUDV # 1–12) scenarios. In the daytime scenarios the paired Mann–Whitney U tests found statistically significant differences for the dominance metric (*p* = 0.000), the level being higher in the absence of vegetation than when it was present (mean rank attitude scores of 70.72, and 48.56, respectively). In the daytime scenarios too, the stress-related metric C3-Highbeta was close to significant (*p* = 0.082), with a mean rank attitude score of 38.46 when vegetation was present, and 29.83 when it was absent. Table 4 shows these results.

### 3.3. Analysis of the Impact of PUDVs Based on Profile

Next, differences were examined based on the participants’ age and gender.

#### 3.3.1. Age

For the comparison of the metrics related to the perception of safety between the different PUDVs, the Kruskal-Wallis test is applied because it has more than two categories and the variables do not meet the normality criterion (K-S Test, *p* < 0.05). The Kruskal-Wallis test detected significant differences for the dominance metric (*p* = 0.000), particularly in the night-time scenarios (*p* = 0.000). At night-time, participants over 65 years of age showed lower levels of dominance (mean rank attitude score of 82.07). Significant differences were also detected for the stress-related metric C3-Highbeta (*p* = 0.006) with a mean rank attitude score of 160.50, with an increase in the mean rank attitude score in the age range 36–45 years. This was repeated in the night-time scenarios (*p* = 0.021). In addition, significant differences were found in the representation of objects metric F4-Gamma at night-time, with higher levels from 46 years of age. Table 5 shows these results.

#### 3.3.2. Gender

To compare the metrics related to the perception of safety between the different PUDVs, taking into account that the variables do not meet the normality criterion (K-S Test, *p* < 0.05), the Mann-Whitney test is applied, which has two categories. The paired Mann–Whitney U tests detected significant differences for the dominance metric (*p* = 0.001), with a mean rank attitude score of 194.72 in men and 159.08 in women. It was observed that this difference was evident only in the night-time scenarios (*p* = 0.001), with men feeling greater dominance than women. At the neurophysiological level, differences close to significant (*p* = 0.055) were detected only for the metric related to the representation of objects F4-Gamma, in daytime scenarios, with a higher level in women. Table 6 shows these results. To delve further into these differences, a more detailed analysis of the design elements was carried out, separating the groups by gender.

##### Women

In daytime scenarios the presence of vegetation significantly reduced the feeling of dominance in women (*p* = 0.045). However, this variable did not affect night-time scenarios. Regarding the number of traffic lanes, significant differences were detected at the neurophysiological level for the stress-related metric C3-Highbeta, and in the metric related to the representation of objects, F4-Gamma. Thus, in both daytime and night-time scenarios, more traffic lanes increased stress (*p* = 0.014, *p* = 0.025, respectively). In addition, in daytime scenarios the F4-Gamma metric also increased significantly (*p* = 0.009). Lighting colour temperature had a significant impact on the dominance metric (*p* = 0.001), which showed higher levels with medium-high temperatures (4500–10,500 K).

##### Men

In daytime settings vegetation significantly reduced feelings of dominance in men (*p* = 0.007). It also affected the stress-related metric C3-Highbeta (*p* = 0.013) and the metric related to the representation of objects F4-Gamma (*p* = 0.009). In daytime scenarios the number of traffic lanes reduced the dominance metric (*p* = 0.004) and increased the F4-Gamma metric (*p* = 0.000). In the night-time scenarios, the C3-Highbeta metric (*p* = 0.025) increased. Lighting colour temperature had a significant impact on the dominance metric (*p* = 0.030), with higher levels being shown for the median temperature (4500 K).

### 3.4. Analysis of the Impact of the PUDVs on the Metrics Related to Perceptions of Safety, Based on Day or Night Lighting

Differences in the participants’ behaviour were analysed between the daytime (PUDV # A–D) and night-time (PUDV # 1–12) scenarios, based on a lighting analysis. The paired Mann–Whitney U tests (K-S Test, *p* < 0.05 and with two categories) detected significant differences for the stress-related metric C3-Highbeta (*p* = 0.000) with a mean rank attitude score of 118.66 in the night-time scenario and 71.56 in the daytime scenario. In addition, significant differences were detected for the representation of objects metric F4-Gamma (*p* = 0.049), with higher levels in night-time scenarios (with a mean rank attitude score of 108.37 in the night-time scenario, and 91.27 in the daytime). Table 7 shows these results.

### 3.5. Analysis of Design Guidelines for Urban Intervention

Next, we studied the effect that different interventions had on each of the scenarios.

#### 3.5.1. Daytime Scenarios

The following analysis shows the effect that different interventions had on each of the scenarios. Overall, the Kruskal-Wallis test showed significant differences between the four scenarios. Scenario C (without vegetation, and with fewer traffic lanes) generated a higher level of dominance and lower levels in the stress-related metric C3-Highbeta, and the metric related to the representation of objects, “F4 -Gamma”. On the other hand, scenario B (with vegetation, and with more traffic lanes) generated lower levels in the dominance metric and higher in the C3-Highbeta and F4-Gamma metrics. Table 8 shows these results.

Next, a special analysis was performed. This enabled identification of the effect that particular interventions (e.g., number of traffic lanes and vegetation) can have on given scenarios.

**Figure 5:**
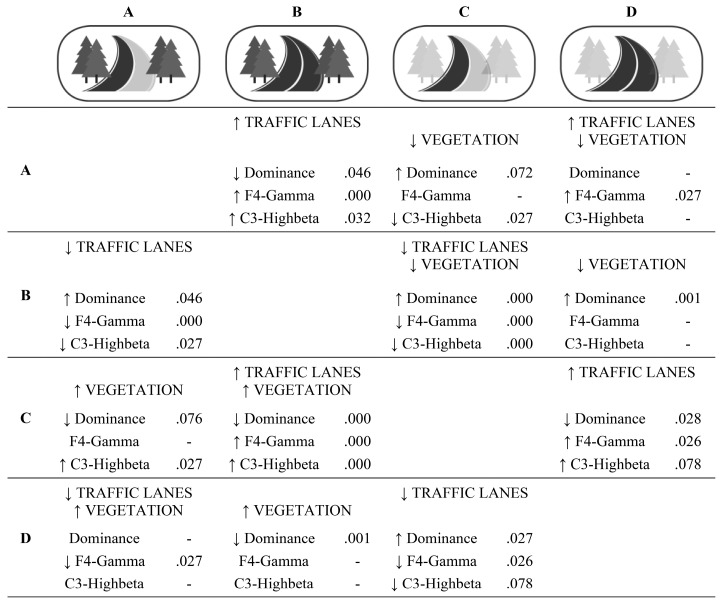
Effect on the metrics of the possible interventions for each of the daytime scenarios.

Figure 5 shows the results of the different interventions and their effects on the psychological and physiological variables. For example, if we start from scenario A (with vegetation, and fewer traffic lanes), more traffic lanes (scenario B) caused a significant deterioration in the dominance metric (*p* = 0.046), and increased the stress-related metric C3-Highbeta (*p* = 0.032) and the metric related to the representation of objects F4-Gamma (*p* = 0.000). This intervention can be applied to each of the crossing scenarios. Thus, in scenario B it is easy to significantly improve the metrics dominance, F4-Gamma, and C3-Highbeta, by reducing the number of traffic lanes (thus moving to stage A) (*p* = 0.046; *p* = 0.000; *p* = 0.032, respectively). A reduction in vegetation (scenario D) also significantly increased the dominance metric (*p* = 0.001). If both interventions are carried out (scenario C), the improvement is synergistic, and all the indicators are significantly improved: dominance (*p* = 0.000) is increased, and F4-Gamma (*p* = 0.000) and C3-Highbeta (*p* = 0.000) are reduced. The same is the case with scenario D, where reducing the number of traffic lanes significantly increases the dominance metric (*p* = 0.028) and reduces F4-Gamma (*p* = 0.026). On the other hand, in scenario C (without vegetation, and fewer traffic lanes), any interventions have negative effects.

#### 3.5.2. Nighttime Scenarios

**Figure 6:**
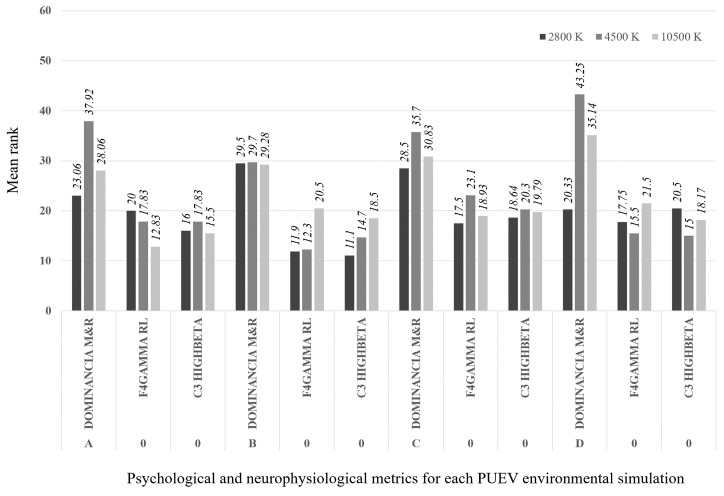
Effect on the metrics of colour temperature modifications for each scenario.

Differences in participants’ psychological and neurophysiological responses to changes in the colour temperature of the illumination in the night-time scenarios were analysed. We started with the daytime scenarios (PUDV # A–D) and tried to identify the best way to illuminate them in terms of colour temperature (Figure 6).

If we look at scenario A (with vegetation, and with fewer traffic lanes), the colour temperature used causes significant differences in the dominance metric (*p* = 0.019). The highest level is generated at a temperature of 4500 K, and the lowest at 2800 K. In the neurophysiological responses, however, scarcely any differences were detected.

If we look at scenario B (with vegetation, and more traffic lanes), there are significant differences for the metric related to the representation of objects F4-Gamma (*p* = 0.050). In this case, the colour temperature to be discarded would be 10,500 K, which significantly increased the level of F4-Gamma, there being no differences in the other two metrics.

In scenario C (without vegetation, and with fewer traffic lanes), lighting colour temperature caused no important differences.

If we look at scenario D (without vegetation, and with more traffic lanes), the result is similar to scenario A. There are significant differences in the level of the dominance metric (*p* = 0.000), the highest level being generated by a temperature of 4500 K, and the lowest by a temperature of 2800 K.

## 4. Discussion

This paper aims to identify design guidelines for urban spaces that might improve the pedestrian’s perceptions of safety; we examined key urban space design variables, such as number of traffic lanes, lighting, and vegetation. These guidelines will be based on both the psychological and neurophysiological responses of pedestrians. The study findings make significant contributions at the methodological and results level.

From the methodological point of view, to the best of our knowledge perceived safety has hitherto been quantified only through self-report. This is not a holistic approach. Up to the present day no neurophysiological metrics have proved capable of quantifying perceptions of safety. However, there are neurophysiological metrics related to unconscious processes that can assess the experience of urban space, for example, sympathetic nervous system activity, stress, and the representation of objects. The most important contribution of the present study is that the quantification of pedestrians’ perceptions of safety was carried out using these metrics. In addition, psychological quantification was performed, using the dominance axis of [37]. Thus, a further key contribution of this article is that these measures were combined to take a more holistic approach to the quantification of the pedestrian’s perceptions of safety. The results obtained show that the psychological and neurophysiological variables used are co-directional and, therefore, may be related.

From the results point of view, the findings of this study provide five important outcomes: (1) impact of design variables on perceptions of safety; (2) differences between daytime and night-time scenarios; (3) combination of design variables; (4) differences based on pedestrians’ demographic profiles; and (5) study of the different actions that pedestrians take.

Regarding the impact of design variables on perceptions of safety, it was found that the selected variables (number of traffic lanes, lighting and vegetation) have an effect at both psychological and neurophysiological levels. It was found that the number of traffic lanes affected almost all the metrics studied: where there are less traffic lanes there are greater feelings of dominance and reduced sympathetic nervous system activity and stress, both in daytime and night-time scenarios. In addition, in daytime scenarios, this reduced problems with the representation of objects. Thus, it was confirmed that number of lanes is a determining and fundamental factor. The colour temperature of lighting only affects dominance. The lowest levels of dominance were associated with lower colour temperatures. These results are consistent with those obtained by [55], also using virtual reality. It should be noted that there are other artificial lighting variables that could have been studied. However, nowadays, new lighting technologies, such as LEDs, are being used for outdoor lighting, replacing traditional lighting systems, often leading to changes in colour temperature [56] that are not always welcomed by the citizenry. This is, thus, a variable of current interest. Above all, the presence of vegetation affects dominance; the suggestion is that its effect may be greater when it is not located in the immediate vicinity of the crossing (especially in daytime scenarios). In general, we conclude that these three design variables, in particular, should be taken into account in the design of urban spaces.

It was confirmed that night-time scenarios generate more difficulty in the representation of objects and cause greater stress than daytime scenarios. These results are consistent with those obtained by [57], who found that daytime scenarios are judged as safer. One possible explanation for this is that daytime vision is a function of the photopic system of cone photoreceptors, and night vision depends on the scotopic system of rod photoreceptors [58]. In the safety context, when someone is asked to judge the characteristics of a lighting installation, it is possible that they look around the site to assess how well they can resolve detail, this being the critical requirement for many tasks. The fovea, which has no rod photoreceptors, is the part of the retina that resolves detail [59]. This confirms that analyses of night and day scenarios must be done separately. It is important to study lighting, since, as we have seen, it is as essential element in the assessment of urban settings [12].

It was found that analyses must consider the compound effect of design variables. Most previous studies have analysed the impact of urban design variables in isolation; for example, number of traffic lanes [29], lighting [60], and vegetation [61]. If the crossing point is a newly created urban space, the variables can be examined individually. However, it is important to keep in mind that existing spaces combine numerous design variables. Thus, in the present study the effect of these design variables was analysed in combination; this showed that design guidelines cannot be universal but depend on a combination of crossing point variables. That is, it is possible that results obtained from individual analyses of variables will not match results obtained when they are combined. For example, vegetation generates different perceptions in the daytime and night-time, depending on the number of existing traffic lanes and the colour temperature of the artificial lighting. This approach would be more useful in the case of urban regeneration, where it is important to check the effect of each of the partial interventions. A combined study provides a deeper analysis. This aspect is a fundamental contribution of this work.

Significant differences were found based on the demographic profile of the participants, that is, their age and gender. Age profile presented significant differences in dominance and stress. As age increases, especially in the group of people over 65, dominance is reduced, and stress levels in night-time scenarios increase. Significant differences were seen in dominance based on gender. Women feel less dominance than men, especially in night-time scenarios. This accords with other studies, where scenarios were rated safer by men than by women [57].

It was shown that there is a need to study certain pedestrian actions, and not just the action of walking, to assess their relationships with perceptions of safety. For example, the intention to cross involves the assessment of many aspects of urban space. In this sense, although many works have analysed the impact of urban design variables on the pedestrian, they have usually focused on their responses while walking on the street, not in crossing situations [24]. The vegetation variable is especially illustrative in this regard. A variety of studies have shown that, compared to environments with little or no natural features, the presence of nature reduces stress and positively influences well-being [18,19]. However, in the present study, it has been shown that the presence of vegetation decreases dominance and increases stress and difficulty in the representation of objects when pedestrians intend to cross a road. This result is consistent with some works [62,63] that have suggested that environments that demand focused or directed attention, such as busy urban spaces, increase stress. Other authors [64,65] have observed that trees, and other aesthetic landscape enhancements at the sides of roads, negatively affect safety by distracting the attention of pedestrians and drivers. Thus, the results of the present work show that it would be interesting to carry out this type of analysis, as pedestrian responses differ, when they are looking at an environment from the enjoyment viewpoint while they are walking, and when they are crossing a street.

## 5. Conclusions

This paper aims to identify urban space design guidelines that will enhance pedestrians’ feelings of safety at street crossings, through measuring participants’ psychological and neurophysiological responses. At the methodological level, the results showed that the psychological and neurophysiological variables used were co-directional. Thus, the EEG metrics studied (C3-Highbeta and F4-Gamma) seem to be good complementary approaches to measurements of dominance related to the urban space experience. As to the design guidelines, the results showed that the impact of design variables depends on whether it is day or night, so it is essential to conduct studies where both situations are taken into account. In general, the pedestrian’s perceptions of safety at street crossings seem to be enhanced by reducing the number of traffic lanes (both in daytime and night-time scenarios), by using a lighting colour temperature of 4500 K, and by reducing surrounding vegetation (especially in daytime scenarios). The combined analysis of design variables showed the need to evaluate the effects that several possible interventions might produce in an existing space. This is essential for urban-planning managers, who must assess different interventions to improve crossing points.

## Figures and Tables

**Table 1:** Statistical treatments.

Analysis and Objective	Statistical Treatment	Expected Outcome
ANALYSIS A Analysis of the level of presence of the environmental simulations of the PUDVs.	Descriptive analysis of means.	Sufficient level of presence.
ANALYSIS B Analysis of the impact of the PUDVs on the metrics related to perceptions of safety.	Statistical techniques comparison metrics related to the perception safety with non-normal distribution (Kolmogorov-Smirnov (K-S) test, *p* < 0.05): Mann–Whitney U (variable = 2 categories) or Kruskal-Wallis (variable >2 categories), between the PUDV of each variable.	Significant differences in the metrics related to perceptions of safety, between the PUDV of each variable.
ANALYSIS C Analysis of the impact of the PUDVs on the metrics related to perceptions of safety, based on participant profile.	Statistical techniques comparison metrics related to the perception safety with non-normal distribution (K-S test, *p* < 0.05): Kruskal-Wallis, for the PUDV of each variable, between age profiles (>2 categories).	Significant differences in the metrics related to perceptions of safety, between the PUDV of each variable, for given age profiles.
	Mann –Whitney U, for the PUDV of each variable, between gender profiles (=2 categories).	Significant differences in the metrics related to perceptions of safety, between the PUDV of each variable, for given gender profiles.
ANALYSIS D Analysis of the impact of the PUDVs on the metrics related to perceptions of safety, based on day or night lighting.	Statistical techniques comparison metrics related to the perception safety with non-normal distribution (K-S test, *p* < 0.05): Mann–Whitney U, between day and night PUDVs (=2 categories).	Significant differences in the metrics related to perceptions of safety, between day and night PUDVs.
ANALYSIS E Analysis of design guidelines for urban interventions.	Statistical techniques comparison metrics related to the perception safety with non-normal distribution (K-S test, *p* < 0.05): Kruskal-Wallis, between the PUDVs (>2 categories).	Design guidelines for urban interventions that use the design variables studied.

**Table 2:** Mann–Whitney U tests for the differences in the mean ranks of psychological and neurophysiological metrics between 1–2 traffic lanes (significance level *p* < 0.05).

Urban Design Variables	Psychological Metrics		Neurophysiological Metrics			
	Dominance		F4-Gamma		C3-Highbeta	
	Mean Rank	*p*	Mean Rank	*p*	Mean Rank	*p*
Traffic lanes						
1	190.86	0.028	94.81	0.073	91.42	0.010
2	166.95		109.61		112.75	
Traffic lanes–Daytime						
1	68.30	0.002	22.07	0.000	27.64	0.006
2	49.06		44.45		40.74	
Traffic lanes–Night-time						
1	124.66	0.478	66.59	0.779	57.10	0.001
2	118.28		68.50		78.88	

**Table 3:** Kruskal-Wallis test for the differences in the mean ranks of the psychological and neurophysiological metrics colour temperature and lighting (significance level *p* < 0.05).

Urban Design Variables	Psychological Metrics		Neurophysiological Metrics			
	Dominance		F4-Gamma		C3-Highbeta	
	Mean Rank	*p*	Mean Rank	*p*	Mean Rank	*p*
Colour temperature						
2800 K	99.03	0.000	63.42	0.631	66.50	0.970
4500 K	144.60		68.40		67.60	
10,500 K	122.61		70.98		68.46	

**Table 4:** Mann–Whitney U tests for the differences in the mean ranks of the psychological and neurophysiological metrics in the absence and presence of vegetation (significance level *p* < 0.05).

Urban Design Variables	Psychological Metrics		Neurophysiological Metrics			
	Dominance		F4-Gamma		C3-Highbeta	
	Mean Rank	*p*	Mean Rank	*p*	Mean Rank	*p*
Vegetation						
No	180.15	0.773	100.97	0.721	109.50	0.103
Yes	179.99		103.92		96.03	
Vegetation–Daytime						
No	70.72	0.000	32.00	0.283	29.83	0.082
Yes	48.56		37.33		38.46	
Vegetation–Night-time						
No	115.82	0.196	65.50	0.508	71.45	0.191
Yes	127.47		69.97		62.63	

**Table 5:** Kruskal-Wallis test for the differences in the mean ranks of the psychological and neurophysiological metrics based on the age of the participants (significance level *p* < 0.05).

Characteristics of the Participants	Psychological Metrics		Neurophysiological Metrics			
	Dominance		F4-Gamma		C3-Highbeta	
	Mean Rank	*p*	Mean Rank	*p*	Mean Rank	*p*
Age						
<25	212.76	0.000	120.96	0.191	118.10	0.006
26–35	163.20		98.82		103.55	
36–45	263.03		97.39		160.50	
46–65	292.50		129.25		54.75	
56–65	230.36		141.36		118.21	
>65	166.33		122.00		119.00	
Age–Daytime						
<25	69.11	0.276	43.28	0.573	43.11	0.369
26–35	55.39		42.38		35.38	
36–45	56.20		33.83		51.83	
46–65	78.50		34.00		31.00	
56–65	93.50		27.00		26.00	
>65	72.30		31.00		38.00	
Age–Night-time						
<25	143.67	0.000	79.72	0.017	77.46	0.021
26–35	113.39		55.64		62.79	
36–45	193.65		63.17		105.50	
46–65	209.50		98.50		28.50	
56–65	146.50		106.30		83.10	
>65	82.07		85.00		86.00	

**Table 6:** Mann–Whitney U tests for the differences in the mean ranks of the psychological and neurophysiological metrics based on the participant’s gender (significance level *p* < 0.05).

Participant’s Gender	Psychological Metrics		Neurophysiological Metrics			
	Dominance		F4-Gamma		C3-Highbeta	
	Mean Rank	*p*	Mean Rank	*p*	Mean Rank	*p*
Gender						
Men	194.72	0.001	97.56	0.269	102.07	0.924
Women	159.08		106.72		102.86	
Gender–Daytime						
Men	59.62	0.422	31.11	0.055	31.89	0.114
Women	54.58		40.15		39.32	
Gender–Night-time						
Men	135.16	0.001	68.05	0.886	69.98	0.518
Women	106.17		67.08		65.64	

**Table 7:** Mann–Whitney U tests for the differences in the mean ranks of the psychological and neurophysiological metrics in daytime and night-time scenarios (significance level *p* < 0.05).

Urban Design Variables	Psychological Metrics		Neurophysiological Metrics			
	Dominance		F4-Gamma		C3-Highbeta	
	Mean Rank	*p*	Mean Rank	*p*	Mean Rank	*p*
Daytime–Night-time						
Daytime	177.55	0.905	91.27	0.049	71.56	0.000
Night-time	178.98		108.37		118.66	

**Table 8:** Kruskal-Wallis test for the differences in the mean ranks of the psychological and neurophysiological metrics in daytime scenarios.

Daytime Scenarios	Psychological Metrics		Neurophysiological Metrics			
	Dominance		F4-Gamma		C3-Highbeta	
	Mean Rank	*p*	Mean Rank	*p*	Mean Rank	*p*
Intervention						
A	59.93	0.000	23.06	0.000	32.17	0.013
B	40.60		46.50		42.50	
C	78.95		20.30		19.50	
D	63.17		40.36		37.21	

## References

[1] Cho G., Rodríguez D.A., Khattak A.J. (2009). The role of the built environment in explaining relationships between perceived and actual pedestrian and bicyclist safety. Accid. Anal. Prev..

[2] Talavera R., Soria J.A., Valenzuela L.M. (2014). La calidad peatonal como método para evaluar entornos de movilidad urbana. Doc. Anàlisi Geogràfica.

[3] Bernhoft I.M., Carstensen G. (2008). Preferences and behaviour of pedestrians and cyclists by age and gender. Transp. Res. Part F Traffic Psychol. Behav..

[4] Liu J.Y. (2015). Fear of falling in robust community-dwelling older people: Results of a cross-sectional study. J. Clin. Nurs..

[5] Turner S., Fitzpatrick K., Brewer M., Park E.S. (2006). Motorist yielding to pedestrians at unsignalized intersections: Findings from a national study on improving pedestrian safety. Transp. Res. Rec..

[6] Landis B.W., Vattikuti V.R., Ottenberg R.M., McLeod D.S., Guttenplan M. (2001). Modeling the roadside walking environment: Pedestrian level of service. Transp. Res. Rec..

[7] Transport D. (2007). Manual for Streets.

[8] Feliciani C., Gorrini A., Crociani L., Vizzari G., Nishinari K., Bandini S. (2020). Calibration and validation of a simulation model for predicting pedestrian fatalities at unsignalized crosswalks by means of statistical traffic data. J. Traffic Transp. Eng..

[9] Karndacharuk A., Wilson D.J., Dunn R.C. (2014). Safety performance study of shared pedestrian and vehicle space in New Zealand. Transp. Res. Rec..

[10] Knight C. (2010). Field surveys of the effect of lamp spectrum on the perception of safety and comfort at night. Light. Res. Technol..

[11] Greene M., Greene R. Urban safety in residential areas. Proceedings of the 4th International Space Syntax Symposium.

[12] Fotios S.A., Unwin J., Farrall S. (2015). Road lighting and pedestrian reassurance after dark: A review. Light. Res. Technol..

[13] Hidayetoglu M.L., Yildirim K., Akalin A. (2012). The effects of color and light on indoor wayfinding and the evaluation of the perceived environment. J. Environ. Psychol..

[14] Tantanatewin W., Inkarojrit V. (2016). Effects of color and lighting on retail impression and identity. J. Environ. Psychol..

[15] Haans A., Kort Y.A.W. (2012). De Light distribution in dynamic street lighting: Two experimental studies on its effects on perceived safety, prospect, concealment, and escape. J. Environ. Psychol..

[16] Suzer O.K., Olgunturk N., Guvenc D. (2018). The effects of correlated colour temperature on wayfinding: A study in a virtual airport environment. Displays.

[17] Ulrich R.S. (1991). Effects of interior design on wellness: Theory and recent scientific research. J. Heal. Care Inter. Des..

[18] Bratman G.N., Hamilton J.P., Hahn K.S., Daily G.C., Gross J.J. (2015). Nature experience reduces rumination and subgenual prefrontal cortex activation. Proc. Natl. Acad. Sci. USA.

[19] Chang C.Y., Chen P.K. (2005). Human response to window views and indoor plants in the workplace. HortScience.

[20] Van den Berg A.E., Hartig T., Staats H. (2007). Preference for nature in urbanized societies: Stress, restoration, and the pursuit of sustainability. J. Soc. Issues.

[21] Lohr V.I., Pearson-Mims C.H. (2006). Responses to scenes with spreading, rounded, and conical tree forms. Environ. Behav..

[22] Foltête J.C., Piombini A. (2007). Urban layout, landscape features and pedestrian usage. Landsc. Urban Plan..

[23] Smith A.L. (2009). Contribution of perceptions in analysis of walking behavior. Transp. Res. Rec..

[24] Montel M.C., Brenac T., Granié M.A., Millot M., Coquelet C. Urban environments, pedestrian-friendliness and crossing decisions. Proceedings of the Transportation Research Board 92nd Annual Meeting.

[25] Granié M.A., Brenac T., Montel M.C., Millot M., Coquelet C. (2014). Influence of built environment on pedestrian’s crossing decision. Accid. Anal. Prev..

[26] Bergeron J., De Lavalette B.C., Tijus C., Poitrenaud S., Leproux C., Thouez J.P., Rannou A., Auberlet J.M. (2008). Effets des caractéristiques de l’environnement sur le comportement des piétons à des intersections urbaines. Le Piéton et son Environnement: Quelles Interactions? Quelles Adaptations?.

[27] Chu X., Guttenplan M., Baltes M.R. (2004). Why people cross where they do: The role of street environment. Transp. Res. Rec..

[28] Dommes A., Cavallo V. (2011). The role of perceptual, cognitive, and motor abilities in street-crossing decisions of young and older pedestrians. Ophthalmic Physiol. Opt..

[29] Dommes A., Cavallo V., Dubuisson J.B., Tournier I., Vienne F. (2014). Crossing a two-way street: Comparison of young and old pedestrians. J. Safety Res..

[30] Lipovac K., Vujanic M., Maric B., Nesic M. (2013). Pedestrian behavior at signalized pedestrian crossings. J. Transp. Eng..

[31] Foot H.C., Thomson J.A., Tolmie A.K., Whelan K.M., Morrison S., Sarvary P. (2006). Children’s understanding of drivers’ intentions. Br. J. Dev. Psychol..

[32] Papadimitriou E., Yannis G., Golias J. (2009). A critical assessment of pedestrian behaviour models. Transp. Res. Part F Traffic Psychol. Behav..

[33] Ewing R., Handy S. (2009). Measuring the unmeasurable: Urban design qualities related to walkability. J. Urban Des..

[34] Ewing R., Handy S., Brownson R.C., Clemente O., Winston E. (2006). Identifying and measuring urban design qualities related to walkability. J. Phys. Act. Heal..

[35] De Kort Y.A.W., Ijsselsteijn W.A., Kooijman J., Schuurmans Y. (2003). Virtual laboratories: Comparability of real and virtual environments for environmental psychology. Presence Teleoperators Virtual Environ..

[36] Steuer J. (1992). Defining Virtual Reality: Dimensions determining telepresence. J. Commun..

[37] Mehrabian A., Russell J.A. (1974). An Approach to Environmental Psychology.

[38] Bakker I., van der Voordt T., Vink P., de Boon J. (2014). Pleasure, Arousal, Dominance: Mehrabian and Russell revisited. Curr. Psychol..

[39] Gifford R., Hine D.W., Muller-Clemm W., Reynolds D.J., Shaw K.T. (2000). Decoding Modern Architecture: A Lens Model Approach for Understanding the Aesthetic Differences of Architects and Laypersons. Environ. Behav..

[40] Aspinall P., Mavros P., Coyne R., Roe J. (2015). The urban brain: Analysing outdoor physical activity with mobile EEG. Br. J. Sports Med..

[41] Gidlow C.J., Jones M.V., Hurst G., Masterson D., Clark-Carter D., Tarvainen M.P., Smith G., Nieuwenhuijsen M. (2016). Where to put your best foot forward: Psycho-physiological responses to walking in natural and urban environments. J. Environ. Psychol..

[42] Higuera-Trujillo J.L., Llinares Millán C., Montañana i Aviñó A., Rojas J.-C. (2020). Multisensory stress reduction: A neuro-architecture study of paediatric waiting rooms. Build. Res. Inf..

[43] Faul F., Erdfelder E., Lang A.G., Buchner A. (2007). G* Power 3: A flexible statistical power analysis program for the social, behavioral, and biomedical sciences. Behav. Res. Methods.

[44] Tilley S., Neale C., Patuano A., Cinderby S. (2017). Older people’s experiences of mobility and mood in an urban environment: A mixed methods approach using electroencephalography (EEG) and interviews. Int. J. Environ. Res. Public Health.

[45] Slater M., Usoh M., Steed A. (1994). Depth of Presence in virtual environments. Presence Teleoperators Virtual Environ..

[46] Niedermeyer E., da Silva F.L. (2005). Electroencephalography: Basic Principles, Clinical Applications, and Related Fields.

[47] Knyazev G.G., Savostyanov A.N., Levin E.A. (2004). Alpha oscillations as a correlate of trait anxiety. Int. J. Psychophysiol..

[48] Choi Y., Kim M., Chun C. (2015). Measurement of occupants’ stress based on electroencephalograms (EEG) in twelve combined environments. Build. Environ..

[49] Keil A., Müller M.M., Ray W.J., Gruber T., Elbert T. (1999). Human gamma band activity and perception of a Gestalt. J. Neurosci..

[50] Delorme A., Makeig S. (2004). EEGLAB: An open source toolbox for analysis of single-trial EEG dynamics including independent component analysis. J. Neurosci. Methods.

[51] Gudmundsson S., Runarsson T.P., Sigurdsson S., Eiriksdottir G., Johnsen K. (2007). Reliability of quantitative EEG features. Clin. Neurophysiol..

[52] Delorme A., Makeig S., Sejnowski T.J. Automatic artifact rejection for EEG data using high-order statistics and independent component analysis. Proceedings of the 3rd International Workshop on ICA.

[53] Hyvärinen A., Oja E. (2000). Independent component analysis: Algorithms and applications. Neural Netw..

[54] Slater M., Steed A. (2000). A Virtual Presence Counter. Presence Teleoperators Virtual Environ..

[55] Maciejewski W.F. (2018). Using Virtual Reality in Quantifying the Relation between Colour Temperature of Public Lighting and Perceived Personal Safety.

[56] Fotios S.A., Yao Q. (2018). The association between correlated colour temperature and scotopic/photopic ratio. Light. Res. Technol..

[57] Loewen L.J., Steel G.D., Suedfeld P. (1993). Perceived safety from crime in the urban environment. J. Environ. Psychol..

[58] Rea M.S., IESNA (1993). The IESNA Lighting Handbook: Reference and Application.

[59] Boyce P.R., Eklund N.H., Hamilton B.J., Bruno L.D. (2000). Perceptions of safety at night in different lighting conditions. Light. Res. Technol..

[60] Peña-García A., Hurtado A., Aguilar-Luzón M.C. (2015). Impact of public lighting on pedestrians’ perception of safety and well-being. Saf. Sci..

[61] Fitzpatrick C.D., Harrington C.P., Knodler M.A., Romoser M.R. (2014). The influence of clear zone size and roadside vegetation on driver behavior. J. Saf. Res..

[62] Kuo F.E. (2001). Coping with poverty: Impacts of environment and attention in the inner city. Environ. Behav..

[63] Mulckhuyse M., Theeuwes J. (2010). Unconscious attentional orienting to exogenous cues: A review of the literature. Acta Psychol..

[64] Fitzpatrick C.D., Samuel S., Knodler M.A. (2016). Evaluating the effect of vegetation and clear zone width on driver behavior using a driving simulator. Transp. Res. Part F Traffic Psychol. Behav..

[65] Mok J.H., Landphair H.C., Naderi J.R. (2006). Landscape improvement impacts on roadside safety in Texas. Landsc. Urban Plan..

